# Evaluation of walking activity data during pregnancy as an indicator of pregnancy loss in dairy cattle

**DOI:** 10.3168/jdsc.2022-0304

**Published:** 2022-12-14

**Authors:** C.P.J. Chen, G. Ferreira

**Affiliations:** School of Animal Sciences, Virginia Tech, Blacksburg 24060

## Abstract

A pregnancy loss or abortion can be assumed when a dairy cow that has been previously diagnosed pregnant shows signs of estrus. In herds using leg-based pedometers as a tool to detect cows in estrus, a sudden increase in walking activity (hereafter, activity peaks) relative to a certain threshold activity triggers an estrous alert that can be confused with a pregnancy loss. The objective of this study was to determine whether pregnant cows can show activity peaks as measured by pedometers. We used data from a dairy herd of 250 milking cows using pedometers as a means of measuring walking activity to detect cows in estrus. Two databases were used in this study, which included the walking activity of the entire herd recorded by the pedometers from January 1, 2018, to December 31, 2021 (database 1), and the calving dates, the insemination dates, the dates when a pregnancy diagnosis was declared pregnant, the dates when a pregnancy diagnosis was declared not pregnant or open, and the abortion dates (database 2). Activity peaks were identified within an experimental unit, which was defined as pregnant cows showing an insemination event followed by a confirmed pregnancy and subsequent calving. The activity peaks were identified using the peak searching algorithm that compares the step count on a given day with the step counts of its adjacent days. The candidate peaks were characterized for their magnitudes by the prominence metric. A chi-squared test was performed to test the specificity of the system. From the 4-yr database, 537 pregnancies or experimental units were identified, and 77 pregnancies showed 1 or more peaks, which means that 14.4% of the pregnancies showed activity peaks. Within the pregnancies showing peaks (n = 77), the median equaled 1 peak/pregnancy, the average equaled 1.53 peaks/pregnancy, and the maximum equaled 13 peaks/pregnancy. In conclusion, activity peaks can be observed for pregnant cows using pedometers. These peaks could generate false estrous alerts during the pregnancy period when using pedometers, and these false alerts should not be interpreted as pregnancy losses.

## Highlights

• Sudden increases of walking activity can be observed for pregnant cows using pedometers.
• Sudden increases of walking activity could generate false estrous alerts in pregnant cows.
• Estrous alerts in cows previously identified as pregnant should not be assumed to indicate a pregnancy loss.

## Graphical Abstract

**Figure:**
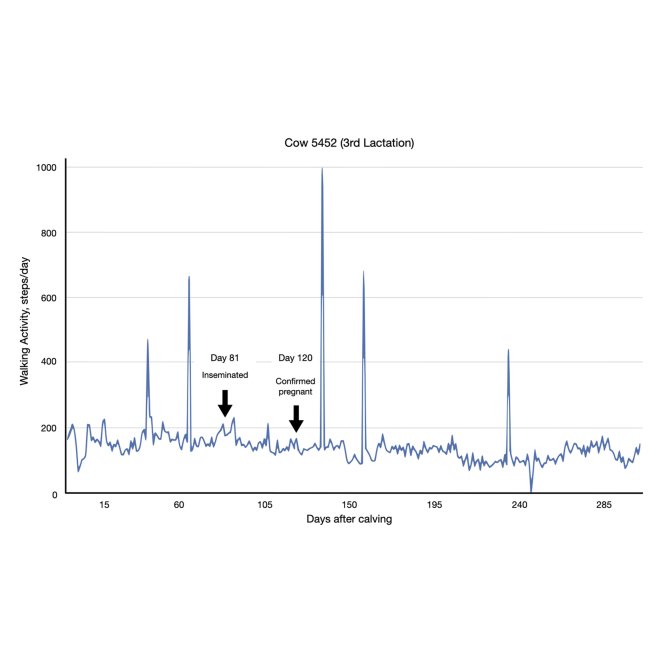
**Summary:** A pregnancy loss or abortion can be assumed when a dairy cow that has been previously diagnosed as pregnant shows signs of estrus. These signs could generate false estrous alerts during the pregnancy period when using pedometers, and these false alerts should not be interpreted as pregnancy losses.

The 21-d pregnancy rate (**PR**) is considered a key performance indicator when evaluating the reproductive performance of the herd (Ferguson and Galligan, 1999; Ferreira, 2013). The PR is a function of the estrus detection rate and conception rate, 2 activities related to breeding (Ferguson and Galligan, 1999). From a management perspective, a high PR indicates that cows are becoming pregnant and, therefore, that replacements for the herd are theoretically secured. However, a high PR might not secure replacements if a high pregnancy loss or abortion rate exists in the herd (Bamber et al., 2009), the latter being defined as the number of pregnancies lost divided by the total number of pregnancies diagnosed within a given period of time. As an example, a herd with a 28% PR and a 22% abortion rate will result in an adjusted PR equal to 22% [i.e., 28% × (100 − 22)% = 22%], whereas a herd with a 24% PR and a 9% abortion rate will also result in an adjusted PR equal to 22% [i.e., 24% × (100 − 9)% = 22%]. Although it has a less effective breeding program, the latter herd has a similar reproductive outcome as the former herd.

In a dairy farm, a pregnancy loss or abortion can be routinely assumed when a dairy cow that has been previously diagnosed as pregnant shows signs of estrus. In herds using leg-based pedometers as a tool to detect cows in estrus (Short et al., 2011; Roelofs et al., 2017), an increase in walking activity (hereafter, activity peaks) relative to a certain threshold activity triggers an estrous alert. Roelofs et al. (2017) evaluated 2 estrus detection systems and reported that the detection of estrus by monitoring walking activity through pedometers has a sensitivity between 63 to 89%, which indicates that some estruses are not detected, and a positive predictive value between 71 to 84%, which indicates that some estrous alerts are false.

To our knowledge, the use of pedometers and the monitoring of walking activity have not been evaluated to detect pregnancy losses in the dairy herd. Conceptually, a pregnant cow is unlikely to show signs of estrus and, therefore, should not show activity peaks unless the cow has aborted. The objective of this study was to determine whether pregnant cows can show activity peaks as measured by pedometers. We hypothesized that pregnant cows do not show activity peaks.

We challenged this hypothesis using data from a dairy herd of 250 milking cows that reported high abortion rates (Table 1; Bamber et al., 2009) and using AfiAct II leg-based pedometers (Afimilk) as a means of measuring walking activity. The farm milks pure Holstein (rolling herd average = 13,291 kg/yr) and Jersey (rolling herd average = 9,139 kg/yr) cows (~75% and 25% of the herd, respectively). The voluntary waiting period lasts 78 to 85 d, the time at which cows receive fixed-time AI after receiving a Double-Ovsynch protocol (Giordano et al., 2012). Pregnancy diagnosis occurs at 32 d and 60 to 74 d postinsemination (first diagnosis and confirmation, respectively) and is performed using ultrasound (Easi-Scan, IMV Imaging) with a 7-MHz probe. Cows previously diagnosed pregnant that show heat or an activity peak are not bred without a previous confirmation of a pregnancy loss. If confirmed open and in good reproductive condition, then cows are subjected to a Resynch protocol (Giordano et al., 2012). Given the cows were managed according to the “Guide for the Care and Use of Agricultural Animals in Research and Teaching” (FASS, 2020) and no human intervention occurred, this retrospective study did not require Institutional Animal Care and Use Committee approval.

**Table 1:** Reproductive summaries (12-mo periods)^1^ of the 250-milking cow dairy used in this study

Item	2020	2021	2022
Heat detection rate, %	57.0	55.6	51.5
Conception rate, %	45.1	49.0	52.4
Pregnancy rate, %	25.2	26.8	26.7
Abortion rate, %	17.2	20.5	20.2

1 Summaries were obtained from report #126 from PCDART (Dairy Records Management Systems) on April 1 of each year.

Two databases were used in this study. The first database included the walking activity of the entire herd recorded by the pedometers from January 1, 2018, to December 31, 2021. The second database included the calving dates, the insemination dates, the dates when a pregnancy diagnosis was declared pregnant, the dates when a pregnancy diagnosis was declared not pregnant or open, and the abortion dates. The former database was obtained from the Afimilk technical staff, whereas the latter database was obtained utilizing the *Activity Tracker* tool of PCDART (Dairy Records Management Systems, Raleigh, NC).

In this study, activity peaks were identified within an experimental unit, which was defined as pregnant cows showing an insemination event followed by a confirmed pregnancy and a subsequent calving 275 ± 7 d afterward. Pregnant cows were discarded as experimental units when calving did not follow an insemination event, presumably due to a pregnancy loss or an erroneously confirmed pregnancy. Once the experimental units were defined, the activity peaks were identified using the peak searching algorithm that compares the step count on a given day with the step counts of its adjacent days. The candidate peaks were characterized for their magnitudes by the prominence metric, which was implemented in the Python library, SciPy (Virtanen et al., 2020). The peaks were then filtered by 3 criteria. The first criterion intends to select peaks with outstanding prominence, which share the exact definition of outliers in box plots (McGill et al., 1978). Briefly, in the same individual, any peak with a prominence greater than the 75th percentile plus 1.5 times of the interquartile range of the step counts was kept as candidate peaks. The second criterion was set to exclude high-prominence peaks caused by drifts of the walking activity baseline. A median of step counts in a 10-d adjacent window of the given day was computed. The examined peak must have had a prominence 5 times greater than the median to be further considered a candidate peak. The third and last criterion intended to remove a peak lasting overnight, as estrous signs are unlikely to last more than 24 h (Mattoni et al., 1988; Reith and Hoy, 2018). Any peak that met the 3 criteria was selected and defined as an activity peak (i.e., sudden increase of walking activity). In addition, to discard the possibility that cows' routines have been disturbed by occasional occurrences (Roelofs et al., 2017), peaks were discarded if they occurred on days of chore events, such as hoof trimming, and pregnancy checks, changes of pens, or vaccinations.

Based on our hypothesis, an activity peak during a pregnancy was unexpected. This hypothesis leads to 2 plausible and binary outcomes: pregnancies without peaks and pregnancies with peaks. A chi-squared test was performed using the FREQ procedure of SAS (SAS version 9.4, SAS Institute Inc.) to test the specificity of the system, which is defined as the proportion of pregnancies not showing activity peaks (*p*). Under our hypothesis, the specificity of the system equals 100% (H_0_: *p* = 1).

From the 4-yr database, 545 pregnancies or experimental units were identified, of which 8 were discarded for being too short (<240-d pregnancies) or too long (>300-d pregnancies). The remaining pregnancies lasted 275 ± 7 d. Within the 537 pregnancies analyzed, 77 pregnancies showed 1 or more peaks, which means that 14.4% of the pregnancies showed activity peaks. Within the pregnancies showing peaks (n = 77), the median equaled 1 peak/pregnancy, the average equaled 1.53 peaks/pregnancy, and the maximum equaled 13 peaks/pregnancy (Table 2). On average, the activity peaks were observed 103 ± 71 d after the conception date.

**Table 2:** Pregnancies and walking activity peaks as measured by pedometers over a 4-yr period in a 250-cow dairy farm

Item	Value
Total pregnancies, count	537
Pregnancies showing activity peaks, count	77
Total activity peaks identified, count	118
Average number of peaks, peaks/pregnancy	1.53
Median number of peaks, peaks/pregnancy	1
Maximum number of peaks, peaks/pregnancy	13

Contrary to our hypothesis, pregnant cows can show activity peaks (Figure 1). This means that the specificity (*p*) of the pedometers to show no activity peak for pregnant cows is 85.6% and different from 100% (*P* < 0.01). This specificity implies that sudden increases of walking activity for cows previously diagnosed as pregnant are unrelated to estrous activity and should not be interpreted as pregnancy losses. In this regard, the occurrence of nonspecific estrous alerts has been reported previously for pedometers (Roelofs et al., 2017).

**Figure 1:**
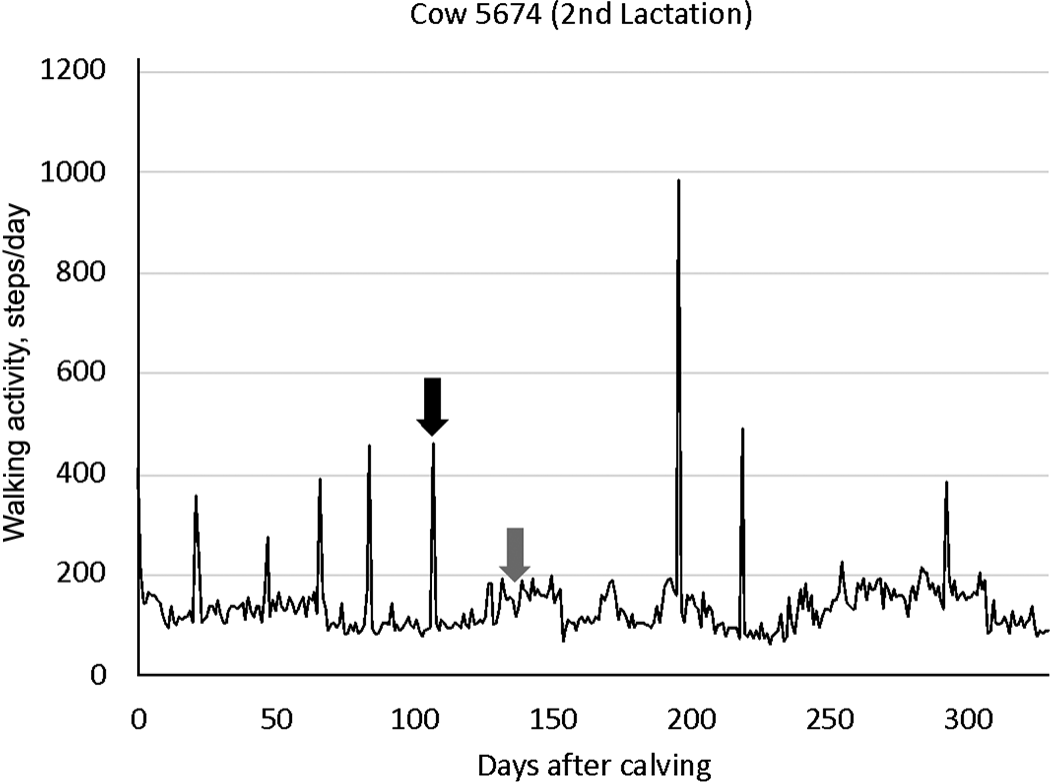
Walking activity of a cow from calving until dry-off. The depicted cow was inseminated on d 106 after calving (black arrow) and was confirmed pregnant on d 138 after calving (gray arrow). Sudden walking activity peaks were observed despite the pregnant status of the cow.

In conclusion, activity peaks can be observed for pregnant cows using pedometers. These peaks could generate false estrous alerts during the pregnancy period when using pedometers, and these false alerts should not be interpreted as pregnancy losses.
